# Total Energy Expenditure, Body Composition, Physical Activity, and Step Count in Japanese Preschool Children: A Study Based on Doubly Labeled Water

**DOI:** 10.3390/nu12051223

**Published:** 2020-04-26

**Authors:** Yosuke Yamada, Hiroyuki Sagayama, Aya Itoi, Makoto Nishimura, Kaori Fujisawa, Yasuki Higaki, Misaka Kimura, Yoshiko Aoki

**Affiliations:** 1 National Institute of Health and Nutrition, National Institutes of Biomedical Innovation, Health and Nutrition, Tokyo 162-8636, Japan; 2 Institute for Active Health, Kyoto University of Advanced Science, Kyoto 621-8555, Japan; 3 Faculty of Health and Sport Sciences, University of Tsukuba, Ibaraki 305-8577, Japan; 4 Department of Health, Sports and Nutrition, Faculty of Health and Welfare, Kobe Women’s University, Hyogo 650-0046, Japan; 5 Faculty of Education, Bukkyo University, Kyoto 603-8301, Japan; 6 Kyoto Compulsory Education School Attached to Kyoto University of Education, Kyoto 603-8163, Japan; 7 Fukuoka University Institute for Physical Activity and Faculty of Sports and Health Science, Fukuoka University, Fukuoka 814-0180, Japan; 8 Faculty of Health and Medical Sciences, Kyoto University of Advanced Science, Kyoto 621-8555, Japan

**Keywords:** doubly labeled water, total energy expenditure, physical activity level, accelerometer, pedometer, fat-free mass, percent fat, step count, estimated energy requirement

## Abstract

Adequate energy intake is essential for the healthy development of children, and the estimated energy requirement of children is determined by total daily energy expenditure (TDEE) and energy deposition for growth. A previous study in Japanese tweens indicated that TDEE could be estimated by fat-free mass (FFM) and step count. The aims of this study were to measure TDEE in Japanese preschool children and to confirm whether TDEE can be estimated by FFM and step count in preschool children. Twenty-one children aged 4–6 years old (11 girls and 10 boys; age, 5.1 (0.9) years; height, 107.2 (6.6) cm; weight, 17.5 (1.7) kg; BMI, 15.3 (1.3); mean (SD)) participated in this study. FFM and 7-day TDEE were obtained by doubly labeled water (DLW). Participants wore accelerometers during the DLW measurement period. No significant differences were observed in age-adjusted height, weight, BMI, FFM (13.0 (1.5) kg), or TDEE (1300 (174) kcal/day) between girls and boys. Girls had significantly higher percent fat and a lower daily step count than boys. Stepwise regression analysis revealed that FFM and step count were significant predictors of TDEE; TDEE (kcal/day) = 85.0 × FFM (kg) + 0.0135 × step count (steps/day). This accounted for 74% of TDEE variance. The current study confirmed that FFM and step count are major determinants of TDEE in Japanese preschool children as well as adolescents, although further research is needed to obtain precise equations.

## 1. Introduction

Nutrition-related factors contribute to approximately 45% of deaths in children aged under 5 years (mainly due to undernutrition) as well as malnutrition in all its forms, such as wasting, stunting, and overweight or obesity, interrupting the healthy growth and development of children worldwide [1,2]. The double burden of malnutrition is considered to be one of the greatest global health issues. Stunting and wasting are the result of poor nutrient intake and/or disease; globally, approximately 149 million children and over 49 million children under 5 years old suffer from stunting and wasting, respectively. Overweight and obesity are another emerging face of malnutrition; currently, over 40 million children globally are overweight or obese, an increase of 10 million since 2000 [1]. The first law of thermodynamics indicates that excess energy intake compared with energy expenditure induces weight gain [3,4]. On the contrary, energy deficiency induces stunting and/or wasting during childhood growth, particularly under the conditions of physically active lifestyle and a heavy burden of infectious disease [5]. Therefore, the assessment of today daily energy expenditure (TDEE) is an essential component of clinical nutrition in children. To measure TDEE in a free-living environment, the doubly labeled water (DLW) technique is considered a standard method. However, the DLW method is limited for research purposes because it is very expensive and involves time-consuming analysis. Therefore, to establish prediction equations for TDEE, more convenient and practical variables, such as daily step counts and body size or composition, are needed in the clinical setting.

The current study aimed to investigate TDEE and to establish a prediction equation of TDEE in Japanese preschool children aged 4–6 years old. At present, there are little data available on TDEE of preschool children in Asia. A previous study examined the TDEE of Japanese tweens (10–12 years old) using DLW and found that their TDEE was comparable to the United States and Canada dietary reference intakes after adjusting for age, body weight, and height [6]. In addition, the authors reported that the participants’ TDEE could be predicted by fat-free mass (FFM) and daily step count [6]. We hypothesized that TDEE could be predicted with FFM and daily step count in preschool children as well as tweens, but the prediction equation is slightly different from that used for tweens because the proportions of basal metabolic rate (BMR) and activity energy expenditure against TDEE differ between preschool children and tweens [7,8,9,10]. A previous study underscored that the choice of pedometer had a substantial impact on step counts in preschool children [11]. Therefore, we conducted an additional experiment to compare pedometers in our study cohort.

## 2. Materials and Methods

### 2.1. Participants

We recruited 23 healthy preschool children aged 4–6 years old from one kindergarten in an urban area in Osaka city, Osaka prefecture, Japan. One child had a fever and canceled their appointment, and one child was unable to completely ingest the DLW. Therefore, we analyzed data from 21 children (11 girls and 10 boys). The measurements were taken in December 2014. Climate statistics during the study period were acquired from the official Japanese government website (Japan Meteorological Agency). The inclusion criteria were healthy subjects without acute or chronic illness, with informed consent to participate in the study obtained from their parents. The experimental protocol was compliant with the Declaration of Helsinki and conducted with the approval of the ethics committee of Bukkyo University (H26-45) and Kyoto Prefectural University of Medicine (E-382). Body weight was measured using an electronic scale to the nearest 0.1 kg with the participants dressed in light clothing. Barefoot standing height was measured to the nearest 0.1 cm using a stadiometer. The BMI percentile was not an exclusion criterion in this study. Overweight and obesity were determined using the international definitions for childhood obesity developed in a workshop organized by the International Obesity Task Force (IOTF) [12]. The IOTF used six nationally representative growth studies and constructed BMI growth curves such that the curves at 18 years of age pass through BMI cutoff points of 25 and 30 for adults [13]. Thinness, which was determined using a previous study that used the same dataset from IOTF, was defined as the curve passing through a BMI of 18.5 at age 18 [14].

### 2.2. TEE and Body Composition Measurement with DLW

TEE was measured for 7 days using the DLW method. Upon arrival at the kindergarten on day 0, a urine sample was acquired for the measurement of baseline ^2^H and ^18^O enrichment before the DLW dose. Each participant drank DLW containing a premixed dose of approximately 0.12 g/kg estimated total body water (TBW) of ^2^H_2_O (99.8 at.%, Taiyo Nippon Sanso, Tokyo, Japan) and 2.5 g/kg estimated TBW of H_2_^18^O (10.0 at.%, Taiyo Nippon Sanso) [15]. Urine samples were collected in the morning and evening of the next day (day 1, Dec. 9th) as well as day 8 (Dec. 16th). The urine samples were stored frozen at −15 °C for later analysis by isotope ratio mass spectrometry (IRMS, Hydra 20-20, Sercon Ltd., Crewe, UK) at Fukuoka University Research Institute for Physical Activity (FUIPA). Detailed analysis using IRMS was conducted as previously described [15,16].

The dilution space of ^18^O and ^2^H (No and Nd, respectively) was determined by dividing the dose of the administered tracer by the intercept method using two day 1 samples and two day 8 samples. All samples collected on day 8 exceeded 8‰ from baseline. The Nd/No values of the present study (1.032 ± 0.015, range 1.007–1.055) were similar to those of previous studies [17,18]. Thus, total body water (TBW) (g) was calculated as the average of the value obtained with Nd divided by 1.041 and No divided by 1.007. TBW (mol) was obtained as TBW (g)/18.02. The elimination rate of ^18^O and ^2^H (ko and kd, respectively) was determined, and the carbon dioxide production rate (rCO2) (mol/day) was calculated as 0.4554 × TBW (mol) × (1.007ko − 1.041kd), with the assumption that isotope fractionation applies only to breath water using Equation (A6) by Schoeller et al. [19], with the revised dilution space constant provided by Racette et al. [17]. The rCO_2_ (L/d) was obtained as 22.4 × rCO2 (mol/day). We assumed that the respiratory quotient (RQ) was 0.87 [20], and TEE was calculated using the modified Weir’s equation [21] as follows: TDEE (kcal/d) = 1.1rCO2 + 3.9rCO2/RQ. The quality checklist is described in International Atomic Energy Agency (IAEA) documents [22]. FFM was calculated using TBW with the age-dependent hydration factor of children [23]. Fat mass (FM) and percent fat (%fat) were calculated using FFM and body weight.

The predicted BMR was calculated using the equation of the Recommended Dietary Allowances for Japanese [24] as in previous studies [16], which systematically reviewed the previous BMR measurements in the Japanese population and established equations with age categories from infants to older adults. The physical activity level (PAL) was calculated as TDEE divided by the BMR predicted by the Japanese equation [25,26]. For further analysis, we also calculated BMR from the Schofield equation [27,28].

### 2.3. Step Count Measurement with Accelerometer-Based Pedometers

Daily step counts were monitored by a triaxial accelerometer (Actimarker, Panasonic, Osaka, Japan) [15,29,30,31]. Preschool children have a variety of physical movements and are forgetful and may easily lose or break their pedometers. Therefore, the accelerometers were placed into a small waist pouch (Kid’s SPIbelt^®^, Austin, TX, USA) and immobilized by taping on cardboard in the pocket. This small waist pouch was made for children to carry various medical monitoring devices during their daily living condition. The pocket is expandable, secure, low-profile, and can tightly hold medical monitoring supplies, such as an insulin pump, continuous glucose monitoring, or EpiPens close to their hips without tangling or bouncing. The pouch is very light and has an adjustable strap for the waist circumferences for children. Its elastic belt fits waist size of 46 to 68 cm. The pocket size is 3 cm x 14 cm. The fastener of the pouch was locked by researchers and children and parents could not access and manipulate the accelerometers during the measurement. We confirmed that the accelerometers tightly contacted their hip. We explained the importance to fasten the belt of the waist pouch to tight around subject’s waist to their parents and kindergarten teachers, and let them to check the tightness regularly for the length of the study. We had compared our current method to simply wearing the accelerometer on an elastic band that ensures that it is tight against the hips of preschool children. No statistical difference was found between step counts of an Actimarker simply worn on an elastic band against the hip and that of an Actimarker placed in the SPIbelt pouch. The subjects wore the accelerometer from the morning of day 0 to the morning of day 9. The participants were requested to wear these devices at all times, with the exception of special circumstances, such as when dressing, bathing, swimming, and sleeping. We analyzed seven consecutive days’ data (days 1 and 8), excluding the days that subjects started wearing and removed the accelerometers. As in previous studies [11,32], we excluded values below 1000 steps/day and above 30,000 steps/day and treated them as missing data. Two days from one child and one day from two other children were excluded because both Actimarker and Lifecorder recorded <1000 steps/day; the excluded days accounted for 2.7% of the total observed days. We excluded non-valid days based on the previous recommendation [33,34]. Wear time was determined by subtracting non-wear time from 24 h. Non-wear time was defined by an interval of at least 60 convective minutes of zero activity intensity [33], and a recent recommendation of not allowing any interruptions in the child population was followed [34].

### 2.4. Statistical Analysis

The sample size was calculated using multiple linear regression analysis to establish a prediction equation for TDEE with two predictors (FFM and step counts) with an effect size f^2^ of 0.5, α of 0.5, and power of 0.80, requiring a sample size of 18 (G*Power 3.1.9.7, Universität Kiel, Germany). The results are presented as mean ± SD except where indicated. Analysis of covariance (ANCOVA) was used to analyze sex differences, and data were adjusted for age because age is a critical determining factor during the rapid growth phase (from 0 to 6 years old). Simple correlations were conducted using both the Pearson and Spearman methods to confirm the relationship because of the small sample size. To examine factors related to TDEE, partial correlation analysis was applied, with sex, age, height, weight or FFM, and FM or %fat as adjusting variables. To standardize FFM or FM, FFM and FM were treated as covariates because the intercept of the linear regression of TDEE against FFM or FM is significantly different from zero. Multiple linear regression analyses for predicting TDEE, FFM, and daily step counts were entered into the regression equation simultaneously. To confirm the result, stepwise regression analysis was also conducted; age, weight, height, BMI, percent body fat, as well as FFM and step counts were independent variables, and TDEE was the dependent variable, with 0.05 entry and 0.10 removal for the probability of F. Differences between the two accelerometers were analyzed using Pearson correlation and a Bland–Altman plot. The threshold for statistical significance was p < 0.05. SPSS ver. 22 (IBM Inc., Tokyo, Japan) was used for all statistical analyses.

## 3. Results

### 3.1. Climate Statistics during the Study Period

The mean monthly air temperature was 27.8 °C in July and August and 5.8 °C in February for 2014 in Osaka, Japan. During the study period, the mean air temperature was 7.2 °C, the daily maximum temperature was 10.4 °C, the daily minimum temperature was 4.3 °C, the mean wind speed was 3.1 m/s, and the relative humidity was 61.6%. Precipitation (rainfall) was countable on two days between Dec 9th and 16th, 2014. The latitude of Osaka is 34.7, and the longitude is 135.5; the study region is classified as a humid subtropical climate area.

### 3.2. Physical Characteristics and Sex Difference

Table 1 shows the physical characteristics, body composition, energy expenditure, and daily step counts of the participants. The mean (SD) of TDEE, weight, FFM, and step counts were 1300 (174) kcal/d, 17.5 (1.7) kg, and 13.0 (1.5) kg, respectively. No participants were classified as overweight or obese. One boy and two girls were classified as thin. The mean of TDEE of the three thin children was 1275 (233) kcal/day, and that of the other 18 children was 1305 (170) kcal/day. The mean (SD) of the wear time was 13.3 (1.5) hour/day. The mean (SD) of the step counts recorded by Actimarker and Lifecorder was 14,742 (4083) and 11,547 (3024) steps/day, respectively. The BMR estimated by the Japanese equation was slightly higher than but relatively similar to that of Schofield (3.8%). The BMR estimated by the Japanese equation was highly correlated with that of Schofield (r = 0.907, *p* < 0.001).

### 3.3. Determinants of TDEE

To examine the sex differences of the measured variables, ANCOVA was used with adjustments for age because age is a critical factor for preschool children. The marginal mean and standard error of the mean (SEM) were obtained with age as a covariate (mean age of 5.1 years) and are shown in Table 2. There were no significant differences between girls and boys in height, weight, BMI, FFM, TDEE, estimated BMR, or PAL. Girls had significantly higher FM and %fat and lower step counts than boys.

**Figure 1:**
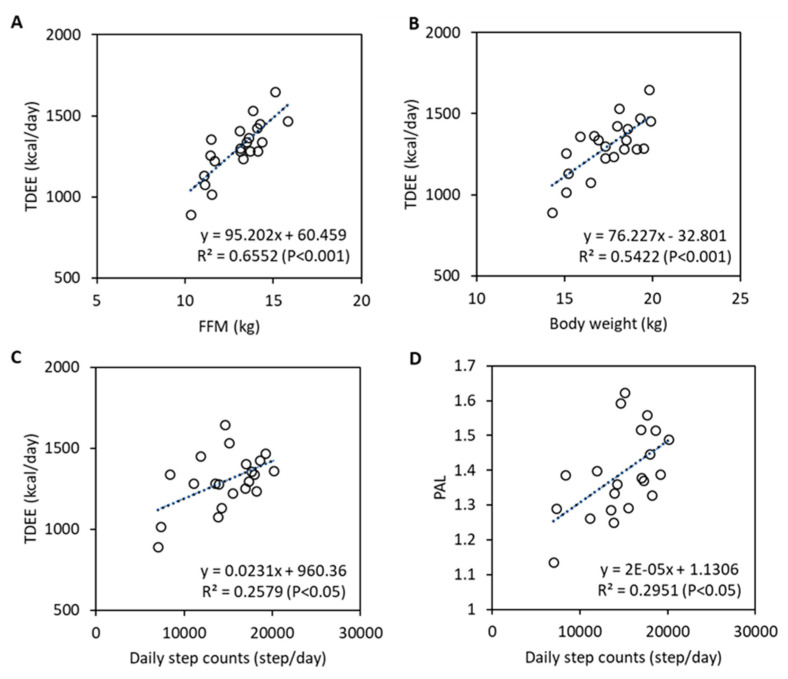
Simple correlation between total daily energy expenditure (TDEE) and fat free mass (**A**), body weight (**B**), or daily step counts (**C**). Relationship between physical activity level (PAL) and daily step counts is also shown in panel (**D**).

Figure 1 shows the relationship between TDEE and FFM, body weight, or step counts, and between PAL and step counts. FFM, body weight, and step counts were significantly correlated with TDEE, and step counts were significantly correlated with PAL both in Pearson and Spearman correlation coefficients. Table 3 shows the partial correlation coefficients between TDEE and the other variables. Height, weight, FFM, and step counts were significantly correlated with TDEE, even after adjusting for sex and age. After adjusting for height as well as sex and age, the weight, FFM, and step counts were still significantly correlated with TDEE. Step counts were still significantly correlated with TDEE, even after adjusting for age, height, weight or FFM, and FM or %fat.

We conducted stepwise multiple linear regression analysis with TDEE as a dependent variable. The potential predictors were sex, age, height, weight, FFM, FM, %fat, and step counts. FFM and step counts were selected as significant predictors of TDEE. Table 4 shows the regression coefficients and their 95% confidence intervals. The equation was TDEE (kcal/day) = 85.0 × FFM (kg) + 0.0135 × step count (steps/day) − 4.7. This accounted for 74% of the TDEE variance. If we used weight instead of FFM, the equation was TDEE (kcal/day) = 70.2 × Weight (kg) + 0.0187 × step count (steps/day) − 204.3. This accounted for 71% of the TDEE variance.

## 4. Discussion

This study aimed to examine TDEE and its determinants in Japanese preschool children aged 4–6 years old. We found that the mean (SD) of TDEE was 1300 (174) kcal/day for the total sample of children. After controlling for age (with a value of 5.1), the marginal means and SEMs of TDEE in girls and boys were 1282 ± 57 and 1320 ± 60 kcal/day, respectively. Simple and partial correlation indicated that FFM and daily step counts were significantly correlated with TDEE, and the correlation was independent of their age, sex, and height. Stepwise regression analysis revealed that FFM and step count were significant predictors of TDEE. Although our study used a small sample size because of the high cost of isotopes and analysis of DLW, the result is important for establishing an accurate estimated energy requirement (EER) for Asian preschool children because, to date, such an analysis has been carried out in only one study (in the Japanese language) so far, which examined the TDEE of eight Japanese preschool children with short stature [35].

The current Japanese dietary reference intake (Japanese-DRI 2020) reviewed DLW studies in preschool children from North America or European countries to tentatively establish EER: the EERs are 1250 and 1300 for 3–5-year-old girls and boys, respectively [10]. In the DRI, the reference height is 103.2 and 103.6, and the reference weight is 16.1 and 16.5 kg in girls and boys, respectively. The present TDEE values are comparable to the current EER and demonstrate the usefulness of the current EER for Japanese preschool children aged around 5 years. A previous study on eight Japanese children with short stature (mean (SD) age, 5.2 (0.5) years; height 96.2 (2.5) cm; 13.4 (1.2) kg) found a TDEE of 1133 (162) kcal/day [35]. This value is lower than that in our current study and the current DRI because the height and weight were lower than reference values. Several studies suggest that there are potential racial/ethnic differences in energy metabolism, body size, and whole-body or organ-specific composition [36,37,38]. At present, we do not have enough data for Asian populations, so further studies are needed.

We found that the FFM and step counts correlated with TDEE, and the relationship was independent of age, sex, height, or %fat. Additionally, TDEE could be predicted by FFM and step counts. Our sample size was small, and the age range was limited, so the contribution of age, sex, or FM to the variance of TDEE is unknown, but the current results indicate the important effects of FFM and physical activity on TDEE. This conclusion is supported by previous research that found that FFM and step counts significantly contributed to the variance of TDEE in Japanese tweens aged 10–12 years [6]. The equation used in the current study was TDEE (kcal/day) = 85.0 × FFM (kg) + 0.0135 × step count (steps/day) for our preschool children, and this equation accounted for 74% of the TDEE variance. The established equation for tweens aged 10–12 years in previous research was TDEE (kcal/day) = 51.1 × FFM (kg) + 0.0505 × step count (steps/day) − 177 [6]. The limited sample size gave us a large 95% CI for the regression coefficients, and this limitation needs some attention. However, the coefficient of FFM was larger for preschool children compared with tweens. There are several possible reasons for these findings: the proportion of “metabolically active organs” which have a higher metabolic rate (such as heart and kidneys, brain, and liver [39,40]) against FFM decreases with growth [41]; the metabolic rate of organs such as the brain also decreases with growth [42,43]. By contrast, the standardized beta of the coefficients of FFM against TDEE is identical between our study and the previous study.

For step counts, the regression coefficients in the preschool children were about one-fourth that in tweens. A potential explanation is that the proportion of activity-based energy expenditure against TDEE is much lower in preschool children aged 4–6 (mean (SD) of PAL, 1.44 (0.15)) than in tweens aged 10–12 (1.60 (0.16) and 1.56 (0.19) in boys and girls, respectively). This phenomenon is confirmed by the description of several national dietary reference intake guidelines [8,9,10]. This may be a potential mechanism for the lower contribution of step counts to TDEE in our preschool children than the previously studied tweens [6]. Although the regression coefficients might be different between age groups, FFM and step counts were still important predictive variables for TDEE in children.

Previous studies have reported that the physical activity of children is different between spring, summer, fall, and winter: children are less active in winter in areas of a particular climate, including but not limited to Edmonton, Norway, South West England, Denmark, and Auckland [44,45,46,47,48]. We conducted our experiment in December in a humid subtropical climate area in which the mean air temperature was 7.2 °C. The experiments of the previous study in Japanese tweens [6] were also conducted in November, November–December, and February in an area with a climate similar to that of the Osaka prefecture. Therefore, we suggest comparing the current result with the previous study to explore seasonal effects on physical activity [6]. Another previous study also suggested that the rainfall decreased daily step counts, although most effects of day length, wind speed, and hours of bright sunshine on step counts were trivial or unclear [47]. However, as the previous study on Japanese tweens [6] did not describe rainfall or other weather information, further studies are needed to evaluate the influence of rainfall.

Estimated TDEE can be provided by Actimarker, and it has been validated against DLW and indirect calorimetry in adult populations [15,30,49]. However, a series of previous studies demonstrated that the relationship between accelerometer outputs and METs, and thus EE, was completely different between preschool children, tweens, and adults [50,51,52]. We, therefore, need to be especially careful if we use accelerometers to acquire data in children. The TDEE values provided by accelerometers are outside the scope of the current purpose as well as the scope of the previous research [6].

The current study has several limitations. In addition to a small sample size, the participants were recruited from one kindergarten in an urban area. There is variation in the obesity or undernutrition status and physical activity levels between rural and urban areas, even in the same country [13]. To generalize these observations, multisite studies are needed. A small sample size may induce unstable results in multiple-variable analysis, and studies with large sample sizes are also needed to confirm the current results. However, we underscore the fact that even with our small sample size, the variables (i.e., FFM and step counts) that were selected as significant predictors for the TDEE of Japanese preschool children were the same as those used in the previous study with Japanese tweens. Another potential influence factor is the choice of accelerometer. The accelerometers were made according as the Japanese Industrial Standards for pedometers (JIS S7200-1993; president of the committee, Dr. Yoshiro Hatano). The accelerometers were tested with an acceleration of 2.4 and 4.9 m/s^2^ induced by the oscillation generator, with a maximum acceptable error of ±3%. However, determining the actual “step” count of preschool children from accelerometer output based on a given algorithm may be complicated. Previous studies have indicated that there is no single objective physical activity assessment instrument that is appropriate for all situations, populations, and research questions [53,54,55]. Further studies are needed to address this issue.

## 5. Conclusions

In conclusion, in our study, TDEE was measured using the DLW method in Japanese preschool children with standard body sizes. These data could be helpful when updating the current Japanese EER in the future. In addition, our study confirmed that the TDEE could be predicted by FFM and step counts in preschool children as well as tweens. The organ-specific metabolic rate or its change during growth and the proportion of activity energy expenditure against TDEE may result in differences in the regression equation between preschool children and tweens.

## Figures and Tables

**Table 1:** Physical characteristics, body composition, energy expenditure, and daily step counts of the participants (*n* = 21).

	Mean	±	SD
Age (year)	5.1	±	0.9
Height (cm)	107.2	±	6.6
Weight (kg)	17.5	±	1.7
BMI (kg/m^2^)	15.3	±	1.3
*n* (%) of overweight or obesity by IOTF (Cole et al. 2000)	0 (0%)		
*n* (%) of thinness by Cole et al. (2007)	3 (14%)		
FFM (kg)	13.0	±	1.5
FM (kg)	4.5	±	0.9
%fat (%)	25.5	±	4.3
TDEE (kcal/day)	1300	±	174
TDEE (kcal/day) for normal BMI group (*n* = 18)	1305	±	170
BMR_Japanese_ (kcal/day)	903	±	50.0
BMR_Schofield_ (kcal/day)	868	±	33.0
PAL	1.44	±	0.15
Step count (step/day) by Actimarker	14,742	±	4083

BMI, body mass index; FFM, fat-free mass; %fat, percent body fat; TDEE, total daily energy expenditure; BMR_Japanese_, predicted basal metabolic rate with the equation for the Japanese population; BMR_Schofield_, predicted basal metabolic rate with the Schofield equation; PAL, physical activity level based on BMR_Japanese_.

**Table 2:** Comparison between girls and boys using ANCOVA adjusted for age.

	Girls (*n* = 11)			Boys (*n* =12)			*p*-Value
	Mean	±	SEM	Mean	±	SEM	
Height (cm)	108.2	±	1.1	106.1	±	1.1	0.26
Weight (kg)	18.0	±	0.5	16.9	±	0.5	0.19
BMI (kg/m^2^)	15.4	±	0.3	15.1	±	0.3	0.49
FFM (kg)	12.9	±	0.4	13.1	±	0.4	0.75
FM (kg)	5.1	±	0.2	3.8	±	0.2	0.002 **
%fat (%)	28.2	±	1.0	22.6	±	1.0	0.002 **
TDEE (kcal/d)	1282	±	57	1320	±	60	0.68
BMR_Japanese_ (kcal/d)	899	±	17.8	907	±	18.8	0.81
BMR_Schofield_ (kcal/d)	852	±	10.5	885	±	11.1	0.07
PAL	1.42	±	0.05	1.45	±	0.05	0.70
Step count (step/d) by Actimarker	12,326	±	1215	17,400	±	1287	0.018 *
Step count (step/d) by Lifecorder	9790	±	898	13,479	±	952	0.020 *

Mean and standard error of the mean (SEM) were estimated as the marginal mean with a covariate appearing in the model at the following value: age = 5.1; BMI, body mass index; FFM, fat-free mass; %fat, percent body fat; TDEE, total daily energy expenditure; BMRJapanese, predicted basal metabolic rate with the equation for the Japanese population; BMRSchofield, predicted basal metabolic rate with the Schofield equation; PAL, physical activity level based on BMRJapanese. *p*-values in bold show significant differences between girls and boys in ANCOVA. * *p* < 0.05, ** *p* < 0.01.

**Table 3:** Partial correlation coefficients between TDEE (kcal/day) and other variables.

Variables	Age	Height	Weight	FFM	FM	%fat	Step Counts
Control Variables							
Sex	0.365	0.650 **	0.767 ***	0.816 ***	−0.007	−0.394	0.626 **
Sex, Age	‒	0.648 ***	0.756 ***	0.804 ***	0.311	−0.190	0.585 **
Sex, Age, Height	‒	‒	0.529 *	0.626 **	0.101	−0.145	0.665 **
Sex, Age, Height, Weight	‒	‒	‒	0.405	−0.405	−0.403	0.567 *
Sex, Age, Height, FFM	‒	‒	‒	‒	0.099	0.097	0.500 *
Sex, Age, Height, FFM, FM	‒	‒	‒	‒	‒	−0.007	0.498 *
Sex, Age, Height, FFM, %fat	‒	‒	‒	‒	‒	‒	0.498 *

TDEE, total daily energy expenditure; FFM, fat-free mass; FM, fat mass; %fat, percent body fat. Step counts obtained by Actimarker. * *p* < 0.05, ** *p* < 0.01, *** *p* < 0.001.

**Table 4:** Multiple linear regression analysis for predicting TDEE (kcal/day) in 4–6-year-old children.

Predictor Variables	B	β	*p*-Value	95%CI for B
FFM (kg)	85.0	0.723	<0.001	(53.6, 11.3)
Step counts (n/day)	0.0135	0.296	0.032	(0.0013, 0.0256)
(Constant)	−4.7		0.98	(−401, 392)

Dependent variable was TDEE, total daily energy expenditure (kcal/day). FFM, fat-free mass; B, unstandardized regression coefficient; β, standardized regression coefficient; CI, confidence interval. Stepwise regression analysis was applied: sex, age, height, weight, fat mass, and percent body fat were not included in the model based on the criteria of 0.05 entry and 0.10 removal for the probability of F. R2 = 0.735 and adjusted R2 = 0.706.
